# Supplementary material for: The Importance of Solution Studies for the Structural Characterization of the Enterovirus 5’ Cloverleaf
Source: Viruses. 2025 Aug 16;17(8):1127. doi: 10.3390/v17081127 (PMC12390691; doi:10.3390/v17081127)
Supplement: Supplementary file 1 [file viruses-17-01127-s001.zip › viruses-3751069-supplementary.pdf]

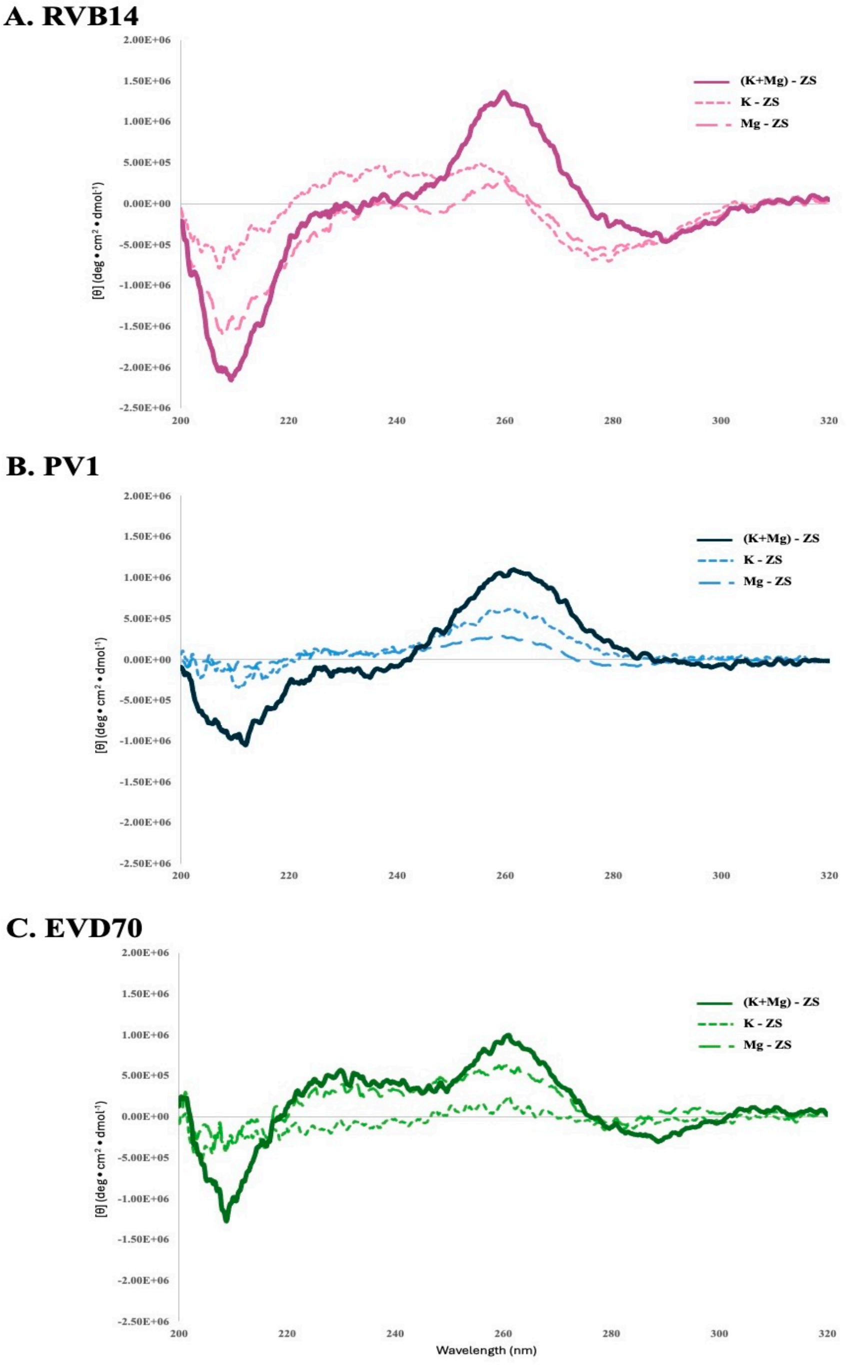

Figure S1: CD difference spectra. For each serotype, the ZS CD spectrum was subtracted from the CD spectrum in each of the ionic environments (K, Mg & K+Mg), in order to further elucidate the change caused by cations. Serotype and ionic conditions are labeled in the Figure. See Table 1 for specific ionic co nnditions. RNA concentration was 0.002 mM.
